# Inadvertent Left Superior Vena Cava Catheterization via the Axillary Vein in a Patient With Persistent Left Superior Vena Cava

**DOI:** 10.1155/carm/9984405

**Published:** 2025-12-07

**Authors:** Angus Hayes, Eric Ho, Sajjad Haider, Elliott Worku

**Affiliations:** ^1^ School of Clinical Medicine, The University of New South Wales, Sydney, New South Wales, Australia, unsw.edu.au; ^2^ Intensive Care Department, Northwest Regional Hospital, Burnie, Tasmania, Australia; ^3^ Intensive Care Service, Royal Prince Alfred Hospital, Sydney, New South Wales, Australia, nsw.gov.au

**Keywords:** axillary vein, central venous catheterization, persistent left superior vena cava, variant anatomy, vascular anomalies, venous access

## Abstract

**Background:**

Persistent left superior vena cava (PLSVC) is a rare congenital vascular anomaly that can complicate central venous catheterization. While axillary vein cannulation is an increasingly recognized alternative to the subclavian or femoral route, inadvertent passage of a catheter into a PLSVC through the axillary vein has not been described in the literature.

**Case Presentation:**

A 46‐year‐old male with refractory status epilepticus required multiple intravenous infusions. Jugular and femoral access was contraindicated due to behavioral risks and local contamination, respectively, prompting ultrasound‐guided catheter placement in the left axillary vein. Initial investigations confirmed venous placement, yet a chest X‐ray suggested an aberrant catheter trajectory near the left hilum. Review of past imaging revealed a PLSVC draining into the coronary sinus, explaining the unusual line path. The malpositioned catheter was removed without incident, and access was successfully re‐established via the internal jugular vein.

**Conclusion:**

This case stresses the importance of recognizing and evaluating unexpected catheter pathways, as variant venous anatomy can complicate central venous access. While routine imaging prior to every cannulation is impractical, prompt investigation of an atypical catheter track is essential to minimize complications. Clinicians should be aware of PLSVC in patients with unusual radiographic findings, including when using the axillary vein for central access.

**Summary**

1. Recognition of variant anatomy
 Clinicians must maintain a high index of suspicion for congenital anomalies like PLSVC whenever a central line shows an unusual course on imaging.
2. Axillary vein cannulation
 The axillary vein can be a safe alternative in specific clinical scenarios, but special caution is warranted if standard insertion sites are contraindicated.
3. Prompt investigation
 In cases of unexpected radiographic line trajectories, a thorough review of prior imaging, possible real‐time modalities (e.g., fluoroscopy and intracavitary ECG), and direct visualization can quickly rule out anatomical variants.

## 1. Introduction

Central venous catheterization is often performed via the subclavian, internal jugular, or femoral veins for administering drugs and fluids, monitoring hemodynamics, and performing other critical interventions. However, contraindications such as patient agitation, site contamination, and anatomical variations occasionally prompt clinicians to choose less common approaches like axillary vein cannulation [1]. Recent evidence suggests that ultrasound‐guided cannulation of the axillary vein can lower the risk of complications like pneumothorax and catheter‐related bloodstream infections [2, 3].

Persistent left superior vena cava (PLSVC) arises from the failure of the left anterior cardinal vein to regress during embryogenesis, resulting in a left‐sided vessel that usually drains into the coronary sinus [4]. Although most patients with PLSVC are asymptomatic, the anomaly can complicate or confuse central venous catheter placement. Several instances of inadvertent PLSVC cannulation have been described via jugular or subclavian access, but the literature does not document this event occurring via an axillary vein approach.

## 2. Systematic Review

A targeted literature search in PubMed and Embase was conducted using the following key terms:

• “Persistent left superior vena cava” OR “PLSVC” OR “left SVC”
• “Central venous catheterization” OR “central venous access”
• “Axillary vein”
• “Misplacement” OR “inadvertent catheterization”

No date or language limits were imposed, and non‐English articles with English abstracts were included. The full PubMed and Embase search strategies are provided in the Appendix. Despite locating reports of PLSVC affecting jugular and subclavian cannulations, we did not identify any previous case reports focusing on inadvertent PLSVC insertion through the axillary vein route. This supports the novelty of our presented case.

## 3. Case Presentation

### 3.1. Patient Background

A 46‐year‐old male with refractory status epilepticus, a history of epilepsy, and intermittent alcohol misuse arrived at our regional emergency department. He presented with ongoing generalized seizures that persisted despite IV benzodiazepines. After intubation and additional loading with levetiracetam, his seizures resolved. However, the patient required prolonged ICU care due to the risk of recurrent convulsions and the need for multiple IV therapies (including antiepileptics and antibiotics).

### 3.2. Rationale for Axillary Vein Access

Both jugular and femoral routes were deemed suboptimal. The patient’s recent violent behavior introduced concerns about line displacement if the jugular vein was accessed, and local contamination risks around the femoral region prompted the team to avoid femoral cannulation. Consequently, an ultrasound‐guided cannulation of the left axillary vein was selected, consistent with literature suggesting safety benefits of this approach [1].

### 3.3. Procedure

**Figure 1:**
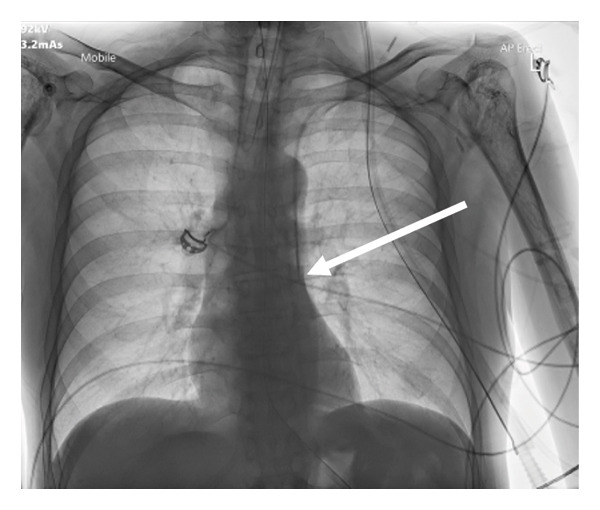
Chest X‐ray demonstrating catheter misplacement. Chest X‐ray shows the central venous catheter coursing along the left mediastinum (white arrows), raising suspicion of placement in the left superior vena cava (PLSVC). Prior CT imaging from 2019 (Figures 2 and 3) confirmed the presence of this anomaly.

Following sterile preparation, a five‐lumen central venous catheter was inserted under ultrasound guidance into the left axillary vein. Dark, nonpulsatile blood was aspirated, and venous blood gas analysis showed a PO_2_ of 44 mmHg, confirming venous placement. However, a postprocedure chest X‐ray revealed the catheter coursing near the left hilum, an atypical path for a standard central venous catheter (Figure 1). An arterial waveform was ruled out by pressure transduction, implying the line remained in a venous structure.

### 3.4. Diagnosis and Management

**Figure 2:**
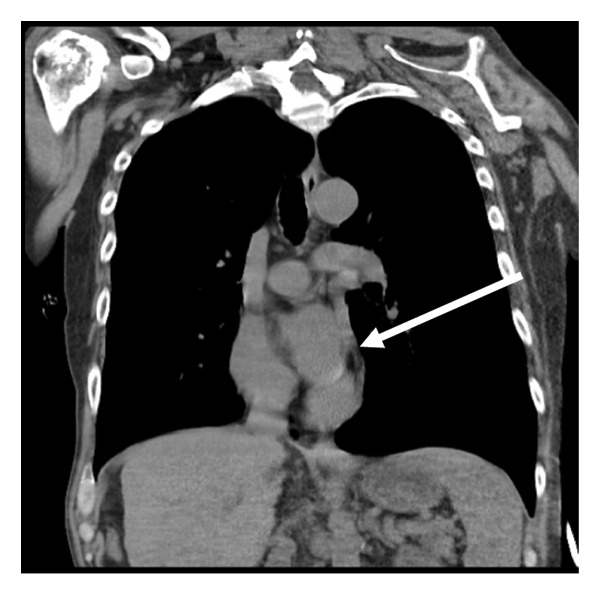
Previous (2019) coronal CT showing the left hilar PLSVC path (white arrow) coursing towards the coronary sinus.

**Figure 3:**
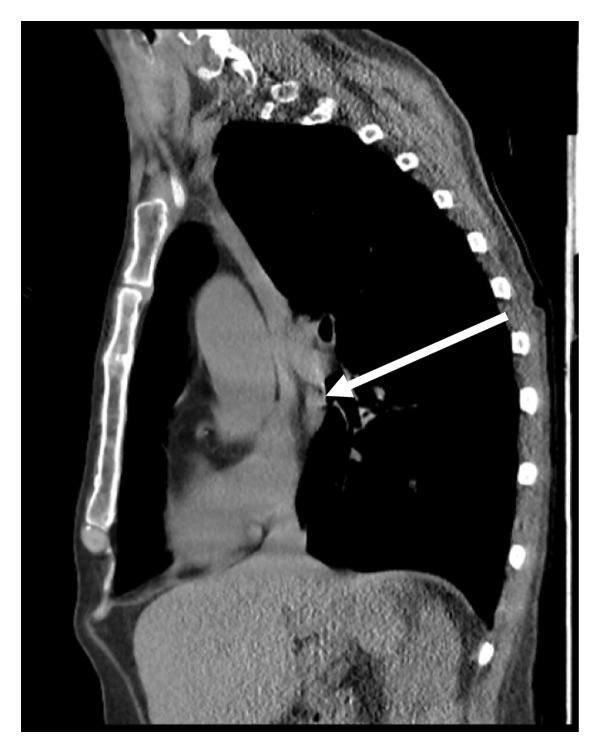
Previous (2019) sagittal CT showing the retroaortic PLSVC path. The PLSVC can be seen extending much further inferiorly than would be expected of an internal jugular vein (white arrow).

A subsequent computed tomography (CT) scan could not fully visualize the catheter tip. However, review of a previous CT from 2019 confirmed the presence of a left‐sided SVC entering the coronary sinus, i.e., a PLSVC, suggesting that the catheter had followed this variant route (Figures 2 and 3). The team removed the malpositioned catheter and replaced it via the internal jugular vein without complications. The patient later self‐discharged against medical advice and was lost to follow‐up.

## 4. Discussion

### 4.1. PLSVC and Clinical Implications

PLSVC occurs in 0.3%–0.5% of the general population and results from an incomplete regression of the left anterior cardinal vein [3, 4]. Frequently asymptomatic, it typically becomes relevant only if encountered incidentally during procedures that rely on standard venous anatomy. The presence of a PLSVC can lead to confusion over arterial versus venous line placement or raise concerns about catheter tip location and potential complications.

### 4.2. Feasibility of Continuing IV Infusions via PLSVC

Although some reports suggest short‐term intravenous therapy through a PLSVC may be safely tolerated—provided the tip is stable, arrhythmias do not occur, and the diameter is sufficient—there is limited consensus regarding extended usage [4]. The risk of delivering irritant infusions into the coronary sinus, as well as the potential for arrhythmias or maldistribution, usually justifies repositioning the catheter into a more standard central vein.

### 4.3. Potential Complications of Ongoing PLSVC Use

1. Arrhythmias: Proximity to the coronary sinus can provoke ectopy or sustained arrhythmias if the catheter tip irritates cardiac tissue.
2. Drug distribution: Infusions delivered into a smaller caliber venous pathway might be less effective or could cause local endothelial irritation.
3. Risk of perforation: Vessel angulation or fragile walls in the PLSVC may predispose to mechanical stress and complications.

### 4.4. Role of Axillary Vein Cannulation

In addition to reducing pleural complications and infection rates, ultrasound‐guided axillary vein cannulation offers a feasible alternative when standard sites are contraindicated [2, 3]. However, identifying a PLSVC via this route relies on vigilance: clinicians must scrutinize any unusual catheter track on chest imaging, especially if the line’s course deviates from the typical right‐sided pathway toward the superior vena cava.

### 4.5. Imaging Recommendations

1. Fluoroscopic guidance: When placing peripherally inserted central catheters (PICCs) or long‐term lines, real‐time radiographic guidance can detect immediate malposition and aid correction.
2. Intracavitary ECG: This helps confirm optimal catheter tip location by recognizing characteristic P‐wave changes as the catheter approaches the right atrium**.**
3. Prior imaging review: Re‐evaluating existing CT or radiographic images may reveal a PLSVC or other vascular variant, expediting the diagnostic process.

### 4.6. Clinical Significance

Our experience highlights that even an approach considered relatively safe (axillary vein cannulation) is not immune to anatomical variations like PLSVC. Recognizing these anomalies reduces the risk of inadvertently classifying a malpositioned catheter as arterial or misdiagnosing complications. For patients who present with challenging behavioral or local site constraints, a high index of suspicion for variant venous anatomy remains crucial.

## 5. Conclusion

This case provides the first documented instance of inadvertent PLSVC cannulation via the axillary vein, underscoring the need for clinicians to remain alert to variant venous anatomies when line placement deviates from the expected route. While comprehensive preprocedural imaging for every patient is impractical, heightened vigilance, rapid assessment of unusual radiographic findings, and consideration of real‐time imaging techniques can prevent delays in care or potential complications. In settings where alternative access routes are required, knowledge of these anatomical variants is the key to achieve safe vascular access.

## Consent

Shortly after extubation, the patient discharged himself against medical advice, making it impossible to obtain written informed consent for publication. In accordance with the Australian National Health and Medical Research Council (NHMRC) *National Statement on Ethical Conduct in Human Research*, we proceeded under the provision that de‐identified clinical data, posing negligible risk to patient privacy, can be published if it confers educational or public health benefit and there is no reasonable avenue to obtain explicit consent. All direct and indirect identifiers have been removed to protect patient anonymity.

## Conflicts of Interest

The authors declare no conflicts of interest.

## Author Contributions

Angus Hayes: conceptualization, methodology, supervision, investigation, writing–original draft, and writing–review and editing. Eric Ho: data curation and writing–original draft preparation. Sajjad Haider: investigation and methodology. Elliott Worku: supervision and writing–review and editing. Sajjad Haider: data curation.

## Funding

No funding was received for this case report.

## Data Availability

All data generated or analyzed during this study are included in this published article (and its supporting information files). No additional datasets were generated. Data sharing is not applicable to this article as no new data were created or analyzed in this study.

## Appendix A: Pubmed Search Terms

(“Persistent Left Superior Vena Cava” [MeSH Terms] OR “PLSVC” OR “left SVC” OR “double superior vena cava” OR “double SVC”) AND (“Central Venous Catheterization” [MeSH Terms] OR “central venous catheter” OR “central venous access” OR “CVAD”) AND (“Axillary Vein” [MeSH Terms] OR “axillary vein access” OR “axillary vein cannulation”) AND (“Inadvertent catheterization” OR “complication” OR “misplacement”).

## Appendix B: Embase Search Terms

(“Persistent left superior vena cava” OR “double superior vena cava” OR “PLSVC” OR “left superior vena cava”) AND (“central venous catheterization” OR “central venous access” OR “central venous catheter” OR “CVAD”) AND (“axillary vein” OR “axillary vein access” OR “axillary vein cannulation”) AND (“inadvertent catheterization” OR “misplacement”) AND [case reports]/lim.
